# 3D-Printed Mesoporous Carrier System for Delivery of Poorly Soluble Drugs

**DOI:** 10.3390/pharmaceutics13071096

**Published:** 2021-07-18

**Authors:** Christos S. Katsiotis, Michelle Åhlén, Maria Strømme, Ken Welch

**Affiliations:** Division of Nanotechnology and Functional Materials, Department of Materials Science and Engineering, Uppsala University, Box 35, SE-751 03 Uppsala, Sweden; christos.katsiotis@angstrom.uu.se (C.S.K.); michelle.ahlen@angstrom.uu.se (M.Å.); maria.stromme@angstrom.uu.se (M.S.)

**Keywords:** 3D printing, fused deposition modelling, FDM, hot-melt extrusion, HME, mesoporous, mesoporous magnesium carbonate, MCM-41, poorly soluble drug, drug delivery

## Abstract

Fused deposition modelling (FDM) is the most extensively employed 3D-printing technique used in pharmaceutical applications, and offers fast and facile formulation development of personalized dosage forms. In the present study, mesoporous materials were incorporated into a thermoplastic filament produced via hot-melt extrusion and used to produce oral dosage forms via FDM. Mesoporous materials are known to be highly effective for the amorphization and stabilization of poorly soluble drugs, and were therefore studied in order to determine their ability to enhance the drug-release properties in 3D-printed tablets. Celecoxib was selected as the model poorly soluble drug, and was loaded into mesoporous silica (MCM-41) or mesoporous magnesium carbonate. In vitro drug release tests showed that the printed tablets produced up to 3.6 and 1.5 times higher drug concentrations, and up to 4.4 and 1.9 times higher release percentages, compared to the crystalline drug or the corresponding plain drug-loaded mesoporous materials, respectively. This novel approach utilizing drug-loaded mesoporous materials in a printed tablet via FDM shows great promise in achieving personalized oral dosage forms for poorly soluble drugs.

## 1. Introduction

A great proportion of newly developed drugs exhibit poor solubility in water [1]. In the Biopharmaceutics Classification System (BCS), these are categorized either as class II compounds—with low solubility and high permeability—or as class IV compounds, with both low solubility and permeability [2]. The low solubility of these drugs consequently leads to low bioavailability with oral administration of the drug and, hence, significant effort has been directed to investigating ways to improve or overcome solubility challenges [3]. Several solutions have been proposed, such as the use of solid dispersions, prodrugs, nanosuspensions, particle size reduction, and complexation [4,5]. A prominent strategy is the amorphization of the drug in a solid dispersion, i.e., the dispersion of the drug in a carrier in the solid state, thus allowing for a faster dissolution, since energy is not required to break the stronger bonds associated with a crystal. Nevertheless, in many cases, such systems are metastable, and recrystallization of the drug may occur [6]. 

Drug incorporation within the pores of mesoporous materials, which can be categorized as a solid dispersion, presents as an appealing alternative technique to overcome the poor solubility of BCS class II and IV drugs, and greatly enhances their release characteristics. Due to the high surface area and narrow pores of the mesoporous materials, recrystallization of the drug is inhibited, and the drug is instead retained in an amorphous state within the pore system [7]. When the amorphous solid dispersion is placed in aqueous media, the drug exhibits an enhanced apparent solubility, which consequently results in a higher drug release. Such drug delivery systems have also been shown to be able to retain their chemical stability over a long period of time [8].

Perhaps the most frequently studied mesoporous material for the drug loading of poorly soluble drugs is mesoporous silica, with one of the specific structures being MCM-41 [7]. Mesoporous silica is synthesized via a templating method, wherein surfactant micelles are covered by silica during the sol–gel process, and are subsequently removed through calcination, thus forming the porous network. In particular, MCM-41 exhibits a hexagonal structure, with the pore width ranging between ~1.5 and 10 nm, depending on the synthesis parameters [9,10,11,12]. MCM-41 has been shown to be able to stabilize drugs in the amorphous form without recrystallization for a significantly long period of time [13,14]. It has been widely studied as a carrier for drug delivery applications, either as synthesized [15,16,17], or following post-synthetic modification [18,19,20,21].

Mesoporous magnesium carbonate (MMC) has been investigated as an alternative mesoporous material for drug delivery. MMC is synthesized in an inexpensive, template-free process using MgO, methanol, and CO_2_ under pressure [22,23]. The synthesized material is amorphous, and formed by the aggregation of smaller nanoparticles [24]. Spaces between the aggregates of nanoparticles in the material give rise to the mesoporous structure. Properties such as specific surface area (~300–800 cm^2^/g) and pore size distribution (2–20 nm) can be tightly regulated by the synthesis parameters [24,25,26]. Magnesium carbonate is ‘generally regarded as safe’ (GRAS) by the FDA, and has demonstrated potential for pharmaceutical applications [25,27,28]. Drug-loaded formulations of MMC have been shown to be able to stabilize the drug in an amorphous state within its pores, whilst remaining chemically stable for a long period of time [26,29]. Moreover, it has also been shown not to induce cytotoxicity [30,31].

The emergence of novel manufacturing techniques in the past decade, such as 3D printing, is addressing the shortcomings and inflexibility of traditional drug development techniques [32]. Fused deposition modelling (FDM) is the 3D-printing technique that has been employed most extensively for pharmaceutical applications [33]. It offers a fast and facile formulation development in a patient-tailored manner. This technology has given rise to drug delivery systems combining multiple active pharmaceutical ingredients (APIs) [34,35], tunable release characteristics [36,37], and formulations intended for various routes of administration [38,39,40]. The tradeoffs of the aforementioned advantages are that thermosensitive APIs are excluded from consideration due to the high temperatures needed during printing, the scalability of the process is problematic, and the selection of pharmaceutical thermoplastic polymers suitable for FDM is limited. 

An additional concern with FDM-produced drug formulations is the long-term stability of the drug in the solid dispersion, i.e., the recrystallization of the amorphous form of the drug [41]. In the case of poorly soluble drugs, instability can be a major factor for the release behavior and overall performance of the final formulation [42]. 3D-printed systems containing poorly soluble drugs have been examined; however, the stability of these formulations has not been evaluated thoroughly. In a recent study, Govender et al. examined this issue, showing good stability with high percentages of drug incorporation, albeit with a polymer insoluble in water [43]. It has also been demonstrated that amorphization via hot-melt extrusion and subsequent FDM can produce formulations in which the drug quickly recrystallizes [44]. These limitations of FDM are starting to be addressed via the use of a combinatorial approach involving novel hybrid systems, comprising one part produced via FDM and another part produced by a different technique to overcome the shortcomings of their respective techniques [45].

In the present study, a combinatorial system is investigated for the successful implementation of a poorly soluble drug in 3D-printed tablets for oral administration. To the best of our knowledge, mesoporous materials have not been used before as poorly soluble drug carriers in 3D printing, targeting the aforementioned benefits. The formulations comprise a drug-loaded mesoporous material, which is 3D printed within a water-soluble polymer matrix. Two distinct mesoporous materials were examined: MMC, and MCM-41. Each one was loaded with celecoxib, serving as a poorly soluble drug model, and extruded with a blend of polyvinyl alcohol—a water-soluble polymer that has been extensively investigated for pharmaceutical applications in 3D printing—and mannitol before being printed via FDM into tablets [37,46]. The resulting formulations were evaluated in terms of their chemical, structural and morphological properties, as well as regarding their in vitro drug release performance. 

## 2. Materials and Methods 

### 2.1. Materials

Mesoporous magnesium carbonate (MMC) was kindly provided by Disruptive Materials AB (Uppsala, Sweden). Celecoxib was purchased from 3Way Pharm Inc. (Shanghai, China). D-mannitol, ethanol absolute >99.8%, ammonia 28%, and tetraethyl orthosilicate 98% (TEOS) were purchased from VWR International (Stockholm, Sweden). Monobasic sodium phosphate, dibasic sodium phosphate, hexadecyltrimethylammonium bromide (CTAB), and polyvinyl alcohol 4-88 (PVA) were all purchased from Sigma-Aldrich (Stockholm, Sweden).

### 2.2. Preparation of Mesoporous Magnesium Carbonate (MMC)

MMC powder with particle sizes of <100 μm was used in this study. The raw MMC was heat-treated in a furnace with a 10-h ramp to 250 °C, and an additional 10-h hold at that temperature, in order to remove any organic intermediate residuals from the synthesis process.

### 2.3. Synthesis of MCM-41

MCM-41 was prepared by adapting a previously reported protocol by Grün et al. [47], following the heterogeneous system synthesis. The molar ratios used were 1 TEOS: 0.152 CTAB: 2.8 NH_3_: 141.2 H_2_O. CTAB was first added to H_2_O and mixed with NH_4_OH. The silica source TEOS was added dropwise to the mixture over 60 min. After an hour of intense mixing at room temperature, the precipitate was filtered and washed with H_2_O. The product was dried overnight at 90 °C and finally calcined in the furnace by heating to 550 °C at 1 °C/min and holding this temperature for 5 h.

### 2.4. Drug Loading of Mesoporous Materials

The same loading procedure and ratio was followed for both MMC and MCM-41, adopting a solvent evaporation method [28]. First, 1 g of celecoxib was dissolved in 300 mL of ethanol, and 10 g of mesoporous material—MMC or MCM-41—was added to the solution. The mixture was left stirring for 48 h at room temperature. Afterwards, the solvent was removed under reduced pressure in a rotary evaporator. Finally, the acquired solid sample was left to dry at 85 °C overnight. Quantification of the drug-loaded mesoporous materials was performed via weighing, thermogravimetric, and gas sorption analysis. 

### 2.5. Gas Sorption Analysis

Gas sorption measurements were carried out using an ASAP 2020 (Micromeritics Norcross, GA, USA). The isotherms for samples of both unloaded and drug-loaded MMC and MCM-41 were recorded at liquid nitrogen temperature (−196 °C) over a relative pressure range (p/p_0_) of 0–1. The samples were first degassed using a Micromeritics Smart VacPrep unit, under dynamic vacuum (1 × 10^−4^ Pa). MMC samples were degassed for 10 h at 85 °C, whereas for MCM-41 samples the temperature was 150 °C for 12 h. Values describing the specific surface area (SSA), pore size distribution, and total pore volume were calculated using MicroActive version 5 software (Micromeritics, Norcross, GA, USA). The SSA was calculated using the Brunauer–Emmett–Teller (BET) model, over the relative pressure range of 0.05–0.25. The pore size distribution was generated using density functional theory (DFT) to analyze the nitrogen sorption isotherms. Compared to methods based on the Kelvin equation, DFT allows for a better estimation of the pore size distribution in micro- and narrow mesoporous materials. Finally, the total pore volume was measured through a single-point adsorption at a relative pressure of p/p_0_ = 0.985.

### 2.6. Filament Preparation

The polymeric filament was prepared via hot-melt extrusion, using a Filabot EX2 single-screw extruder (Filabot Inc., Barre, VT, USA), with a capability of tuning the extrusion screw speed between 0 and 35 rpm. PVA pellets were first ground into smaller particles using a commercial grinder. Mannitol was employed as a plasticizer in high ratios to allow for extrusion after the addition of drug-loaded MMC or MCM-41. PVA, mannitol, and drug-loaded MMC or MCM-41 were mixed with a mortar and pestle before adding the physical mixture to the extruder. The mass ratios used were as follows: (PVA 0.45: mannitol 0.25: loaded MMC 0.3) or (PVA 0.55: mannitol 0.25: loaded MCM-41 0.2). Note that although a filament with 30% content of MCM-41 could be extruded, giving an equivalent mass ratio as with the MMC filament, it was not possible to feed the filament to the printer, thus rendering it unprintable and, therefore, the mass ratio of MCM-41 was reduced to 0.2. In both cases, a total mass of 20 g of the physical mixture was slowly added to the extruder in 5-g portions so as not to overflow the extruder, and extruded at 175 °C through a 1.5-mm-diameter nozzle at a low extrusion screw speed of 12 rpm.

### 2.7. Tablet Printing

Cylindrical tablets of 11-mm diameter and 3-mm height were designed in AutoCAD 2020 (Autodesk Inc., San Rafael, CA, USA). Following the export of the .stl files, PrusaSlicer 2.1.1 (Prusa, Prague, Czech Republic) was utilized to produce the G-codes subsequently loaded onto a Prusa i3 MK3S printer (Prusa, Prague, Czech Republic). Two different infill percentages were prepared for each filament composition: 50 and 70% of grid infill pattern. In both cases, printing was carried out at 205 °C, and the printing bed was set at 40 °C. Masking tape was used to cover the printing area of the bed in order to allow for better adhesion of the object. The tablets were printed with a 0.15-mm layer height, 1 shell perimeter, and no top or bottom horizontal shell layers. A printing speed of 20 mm/s was chosen for the first layer, and 40 mm/s for the subsequent layers. The retraction option in the printer settings was disabled to prevent cracking of the filament, due to its brittle nature, and clogging of the printer’s nozzle.

### 2.8. Attenuated Total Reflectance–Fourier-Transform Infrared Spectroscopy (ATR–FTIR)

ATR–FTIR spectra of the samples were obtained using a Tensor 27 spectrometer (Bruker, Billerica, MA, USA) together with a platinum ATR diamond module (Bruker, Billerica, MA, USA). The spectra were recorded in the range of 400–4000 cm^−1^, at 4 cm^−1^ resolution with 64 scans.

### 2.9. Powder X-ray Diffraction (XRD)

XRD patterns for all samples were acquired from a Bruker D8 Advance Twin/Twin instrument (Bruker, Bremen, Germany) using Cu K-α radiation (λ = 0.154 nm, 45 Kv, and 40 mA). A 2θ range of 2°–80° was used, with a step size of 0.02° and 1 s of measuring time per step.

### 2.10. Differential Scanning Calorimetry (DSC)

DSC measurements were performed on approximately 10-mg samples in perforated aluminum crucibles on a DSC 3+ instrument (Mettler Toledo, Schwerzenbach, Switzerland). The samples were first cooled to −35 °C, and then heated to 300 °C at a rate of 3 °C/min.

### 2.11. Thermogravimetric Analysis (TGA)

TGA was performed using a TGA/DSC 3+ (Mettler Toledo, Schwerzenbach, Switzerland). Samples weighing 15–20 mg were loaded on uncovered alumina pans. They were first treated in a heat cycle under airflow from room temperature to 150 °C and back to room temperature, in order to remove moisture. No other phenomena were observed in this cycle, thus data from these points are not presented in the later graphs. Subsequently, they were heated from room temperature to 700 °C at a rate of 10 °C/min. The thermograms were reported for the final step. 

### 2.12. Scanning Electron Microscopy (SEM)

The morphology of the empty and drug-loaded mesoporous materials, filaments, and tablets was studied with a LEO 1550 SEM (Zeiss, Jena, Germany) operated at an acceleration voltage of 5.0 kV. All samples were first sputter-coated with Au/Pd to reduce charging effects and allow for better imaging.

### 2.13. Drug Solubility Measurement

The solubility of celecoxib in phosphate buffer (0.1 M, pH 6.8) was measured in triplicate. An excess amount of drug (~40 mg) was added to 200 mL of phosphate buffer and stirred for 3 days at 37 °C. Subsequently, samples were removed, filtered with 0.45-μm PTFE filters, and the absorbance at 248 nm was measured with a UV–Vis spectrophotometer (1800, Shimadzu Corporation, Kyoto, Japan) [48]. A calibration curve was prepared using celecoxib dissolved in phosphate buffer with a minimal amount of ethanol to increase the amount of dissolved drug, and measured in the range of 0.1–10 μg/mL (R^2^ > 0.99).

### 2.14. In Vitro Release Studies

The drug release studies were performed according to the USP II method, on a Sotax AT7 (Sotax AG, Aesch, Switzerland). Each vessel contained 900 mL of phosphate buffer (0.1 M, pH 6.8) at 37 °C and stirred at 100 rpm. Aliquots of 3 mL were removed at predetermined time intervals and replenished with the same volume of fresh buffer, maintained at 37 °C. The samples were filtered with 0.45-μm PTFE filters, and the absorbance at 248 nm was measured with a UV–Vis spectrophotometer. The release behavior of 50% and 70% infill tablets of MMC (MMC-T50, MMC-T70) and MCM-41 (MCM-41-T50, MCM-41-T70) was studied and compared to the drug-loaded powders of MMC (MMC-50, MMC-70) and MCM-41 (MCM-41-50, MCM-41-70) that contained celecoxib equivalent to the loading in the corresponding tablets. Additionally, the release of crystalline celecoxib was measured. The amounts of celecoxib in the samples are detailed in Table 1. The values for the tablets were calculated indirectly, based on the mass of the tablets, the percentage of the mesoporous material in each filament—i.e., 30% for MMC and 20% for MCM-41—and, finally, the percentage of celecoxib loaded in the mesoporous materials, i.e., 9% for both. All experiments were performed in triplicate.

## 3. Results and Discussion

### 3.1. Gas Sorption Measurements

**Figure 1:**
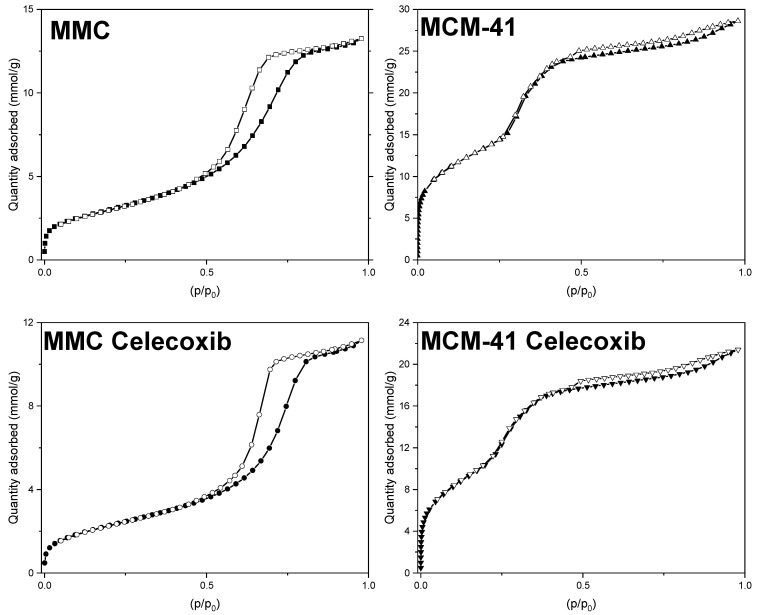
Nitrogen gas sorption isotherms for unloaded and drug-loaded MMC and MCM-41.

**Figure 2:**
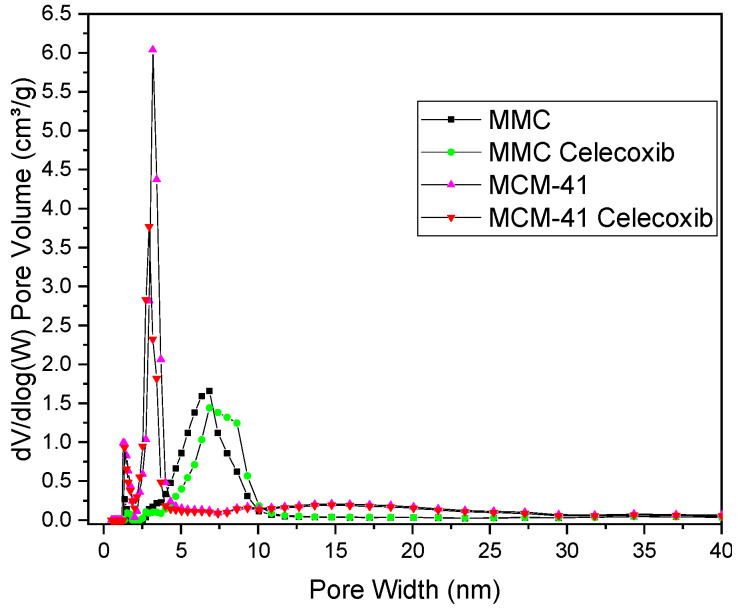
Pore size distribution of unloaded and drug-loaded MMC and MCM-41.

Nitrogen adsorption and desorption curves for loaded and pure MMC and MCM-41 are presented in Figure 1, while values for SSA, pore width, and total pore volume are listed in Table 2. All samples’ isotherms exhibit a typical type IV shape [49], consistent with previous observations regarding MMC and MCM-41 [27,28,47,50]. The shape of the isotherms does not significantly change after loading, which can be explained by both the low loading percentage and the minimal decrease in total pore volume (see Table 2). The pore size distributions for the samples are presented in Figure 2. MCM-41 exhibits a narrow pore size (width) distribution centered at 3.2 nm, and after drug loading we observe an expected reduction in pore volume. Interestingly, although MMC also shows the expected reduction in pore volume after drug loading, the pore size distribution shifts to slightly larger values, whereas the pore size distribution for the loaded MCM-41 sample is shifted slightly to smaller values. For MMC, this increase could be attributed to the shape of the pores, as well as to the manner in which celecoxib fills these pores. Unlike template-based mesoporous materials, such as MCM-41, the pores in MMC particles are proposed to be formed by the spaces between smaller, aggregated nanoparticles [24]. Consequently, the pore size distribution of MMC is broader compared to the templated MCM-41 (see Figure 2). If the smaller pores filled first during the drug loading of MMC, then the pore size distribution would shift to larger values.

The drug loading percentage was calculated based on the measured pore volume reduction and a molar volume of 266.4 cm^3^/mol for celecoxib [51], assuming that the entire decrease of pore volume could be attributed to volume occupied by celecoxib molecules. There is a 0.07 cm^3^/g decrease with MMC, which corresponds to 100 mg of drug per gram of drug-loaded MMC, and a loading percentage of 10%, which is strikingly close to the theoretical loading of 9.1% based on the amounts of celecoxib and MMC used in the loading procedure. Hence, it can be assumed that the drug molecules have been adsorbed within the pore network, and not on the outer surface. Conversely, in the case of MCM-41, there is a 0.22 cm^3^/g post-loading pore volume decrease. This decrease corresponds to 314.9 mg of celecoxib per gram of drug-loaded MCM-41, and a loading percentage of 31.5%, which is much larger than the theoretical loading of 9.1%. A possible explanation for this could be the capillary condensation of drug molecules in pores at a point close to the surface of the MCM-41 particles, effectively blocking portions of the pore from further loading, and also making this empty pore volume inaccessible for nitrogen adsorption during the BET measurement.

### 3.2. Filament Extrusion and Tablet Printing

The filaments and prints in this study are solid dispersions, and the incorporation of non-melting components—i.e., MMC or MCM-41—greatly affected the behavior of the systems during both extrusion and printing. For example, the mesoporous materials tended to make the filament brittle and, thus, in order to produce a filament sufficiently pliable to be printable, a substantial percentage of the plasticizer mannitol was required. An extrusion temperature was selected to allow the polymer and the plasticizer to melt and subsequently drag the solid particles of MMC or MCM-41 along the extruder’s barrel, without the two phases of the physical mixture separating in the process. Since die swell—a phenomenon that has also been previously reported in drug-loaded PVA filaments [46,52]—was observed in both filaments due to the PVA content, a 1.5-mm nozzle was used during the extrusion in order to achieve filaments suitable for the printer, with a diameter as close to 1.75 mm as possible. The extruded filaments’ diameters were measured every 5 cm with a digital caliper, and were found to be 1.60 ± 0.03 and 1.72 ± 0.02 mm for MMC and MCM-41, respectively, see Table 3. The G-code imported to the printer was altered accordingly to account for the different diameters of the filaments.

A print temperature of 205 °C was selected in order to facilitate adequate filament feed through the printer nozzle without causing clogging, which occurred at lower temperatures. Furthermore, the retraction option directs the printer’s extruder to retract the filament a few millimeters after the completion of each layer, so as to avoid leaking as the printhead moves from one end of the object to the other. However, this led to extra movements and strain on an already brittle filament and, thus, the option was disabled, resulting in a minor leaking pattern on all tablets. All tablets were macroscopically observed, and it was noted that they exhibited a well-defined grid infill pattern, with a more densely packed pattern in the 70% infill compared to the 50%. The printing process was found to be highly reproducible for all types of tablets (see Table 3). Interestingly, tablets of different compositions but with the same infill percentage have considerably different weights; this is due to the different ratio of PVA in the two filaments and the higher density of PVA compared to both mesoporous materials. As a result, MCM-41 tablets with identical volume and infill percentage to MMC tablets weigh considerably more, but end up having a similar loading of celecoxib (see Table 1).

### 3.3. ATR–FTIR

**Figure 3:**
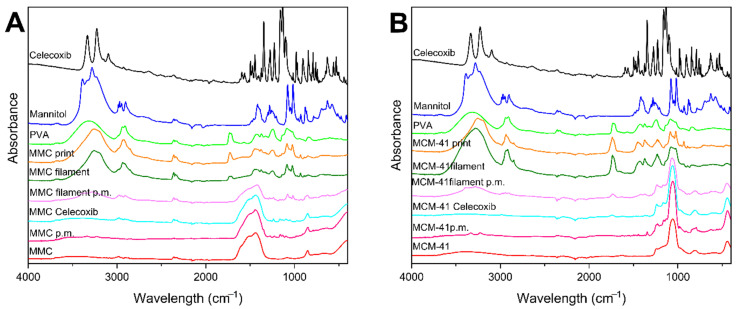
ATR–FTIR spectra of filaments, tablets, and individual components; (**A**) MMC-containing formulations: MMC p.m. is the physical mixture of MMC and celecoxib, MMC Celecoxib is drug-loaded MMC, and MMC filament p.m. is the physical mixture of the filament components; (**B**) MCM-41-containing formulations: MCM-41 p.m. is the physical mixture of MCM-41 and celecoxib, MCM-41 Celecoxib is drug-loaded MCM-41, and MCM-41 filament p.m. is the physical mixture of the filament components.

ATR–FTIR spectra were collected for the individual components used in the filaments/prints, the physical mixture of the mesoporous material and celecoxib, the physical mixture of the components of the filament, the filaments, and the prints, and are displayed in Figure 3. The spectrum for celecoxib exhibited characteristic peaks at 1160 cm^−1^ and 1346 cm^−1^ due to S=O symmetric and asymmetric stretching, respectively. The peak band at 1550–1600 cm^−1^ corresponds to N–H stretching, while the peaks between 3228–3332 cm^−1^ are attributed to NH_2_ stretching [53,54]. The characteristic peaks for PVA at 3330, 2910–2939, 1714–1734, 1425, 1083, and 842 cm^−1^ were observed in its spectrum, corresponding to O–H stretching, C–H stretching, C=O stretching, CH_2_ bending, C–O stretching, and C–C stretching, respectively [55,56]. Peaks observed in the spectrum of mannitol are consistent with previous reports [57,58]. The raw MMC spectrum shows characteristic bands attributed to the carbonate groups at 1442, 1085, and 852 cm^−1^. Moreover, a broad band corresponding to OH is evident at ~3500 cm^−1^ [22,24]. The spectrum of raw MCM-41 indicated characteristic peaks at 802 and 1062 cm^−1^, corresponding to the stretching of Si–O and the vibration of Si–O–Si, respectively, and a broader band at ~3400 due to OH groups [59,60]. The spectra of drug-loaded mesoporous materials exhibit only small differences to their respective spectra when empty. Low-intensity peaks ascribed to the drug can be identified in the fingerprint area < 1500 cm^−1^, which indicates that there are no interactions between the mesoporous materials and the drug, or perhaps that the amount of drug in these samples is below the detection limit of the instrument. The spectra of the physical mixtures of the mesoporous material with the drug show similar results to the drug-loaded materials. ATR–FTIR spectra of samples of the physical mixtures of the filaments, filaments, and prints containing MMC or MCM-41 present no new peak appearances, disappearances, or shifts, and can be generally ascribed to a superposition of constituent peaks.

### 3.4. XRD

**Figure 4:**
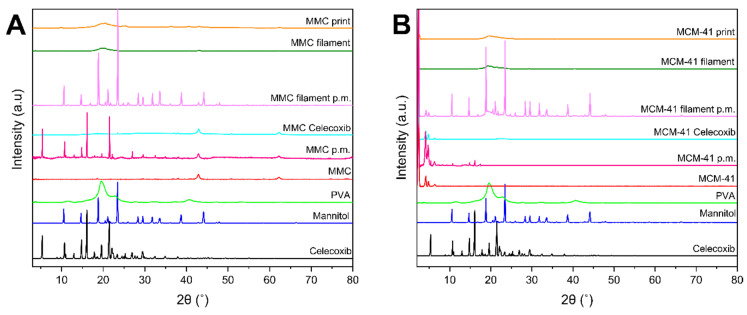
XRD diffractograms of filaments, tablets, and individual components; (**A**) MMC-containing formulations: MMC p.m. is the physical mixture of MMC and celecoxib, MMC Celecoxib is drug-loaded MMC, and MMC filament p.m. is the physical mixture of the filament components; (**B**) MCM-41-containing formulations: MCM-41 p.m. is the physical mixture of MCM-41 and celecoxib, MCM-41 Celecoxib is drug-loaded MCM-41, and MCM-41 filament p.m. is the physical mixture of the filament components.

The diffractograms of celecoxib, excipients, drug-loaded and unloaded mesoporous materials, physical mixtures of mesoporous material and celecoxib, physical mixtures of the filament components, filaments, and prints are depicted in Figure 4. The crystallinity of the drug and mannitol is apparent by the clear sharp peaks of their respective XRD patterns. The patterns of raw MMC are consistent with previous findings, showing MgO peaks at 2θ = 37°, 43°, and 62° [25,27]. The MMC–celecoxib physical mixture diffractogram exhibits crystalline peaks ascribed to celecoxib, as well as the peaks corresponding to MgO at 2θ = 43° and 62°. Drug-loaded MMC exhibits the same peaks as raw MMC, while peaks corresponding to celecoxib are absent, indicating amorphization of the drug loaded within the pores of MMC [28]. The diffractogram of the physical mixture of filament components containing drug-loaded MMC shows crystalline peaks of mannitol, while no other crystalline peaks are apparent; this is expected, as the only other candidate for crystallinity—the drug—is XRD-amorphous in the pores of MMC. The patterns of filament and print of MMC reveal a broad halo at 2θ ~20°, characteristic of amorphous samples. MCM-41 reveals an XRD pattern with peaks in low 2θ values, consistent with previous works [12,61]. The diffractogram of the physical mixture of MCM-41 with the drug presents peaks ascribed to both MCM-41 and celecoxib, with the peaks associated with celecoxib being significantly lower in intensity. A similar behavior is noted for drug-loaded MCM-41 as for MMC, i.e., peaks corresponding to crystalline celecoxib are absent, indicating that the drug after being loaded to the mesoporous material is XRD amorphous. For the physical mixture of filament components containing drug-loaded MCM-41, crystalline peaks corresponding to mannitol and MCM-41 in low 2θ can only be observed for the same reason as in the case of the MMC physical mixture. Furthermore, the MCM-41 filament and print display peaks due to MCM-41 in low 2θ values, and a broader halo at 2θ ≈ 20°, indicating the amorphous nature of the sample.

### 3.5. DSC

**Figure 5:**
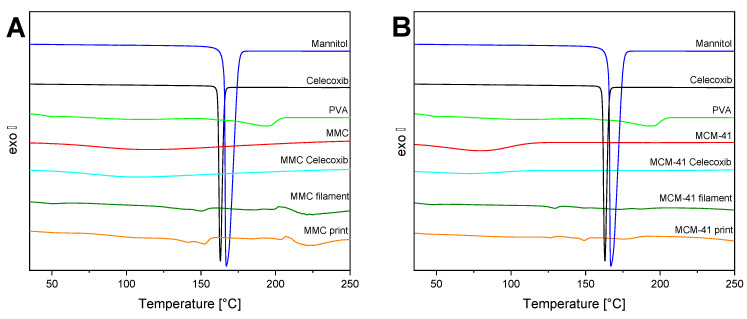
DSC thermograms of filaments, tablets, and individual components; (**A**) MMC-containing formulations; (**B**) MCM-41-containing formulations.

The thermograms of the raw materials, filaments, and prints of both MMC and MCM-41 are depicted in Figure 5. PVA exhibits an endothermic process starting at ~160 °C, corresponding to the melting point at 193 °C. Furthermore, a small peak in the range of 50–60 °C is observed and associated with the T_g_ value [62]. The DSC curve of mannitol displays a sharp endothermic peak ascribed to the melting point at 167 °C [57]. Celecoxib’s melting point is observed very close to that of mannitol, at 162 °C [63]. MMC presents no significant thermal events other than a broad endothermic peak at 75–170 °C, associated with the evaporation of strongly bound water molecules. MCM-41 exhibits the same behavior as MMC, with only a broad endotherm at 50–120 °C. The melting peak of celecoxib is absent in all samples containing drug-loaded MMC and MCM-41, reaffirming the XRD findings regarding the amorphization of the drug within the pores of the mesoporous materials [28]. Finally, for both filaments and prints, the temperature corresponding to the melting point of PVA has been suppressed to ~150 °C, due to the high percentage of the plasticizer mannitol.

### 3.6. TGA

**Figure 6:**
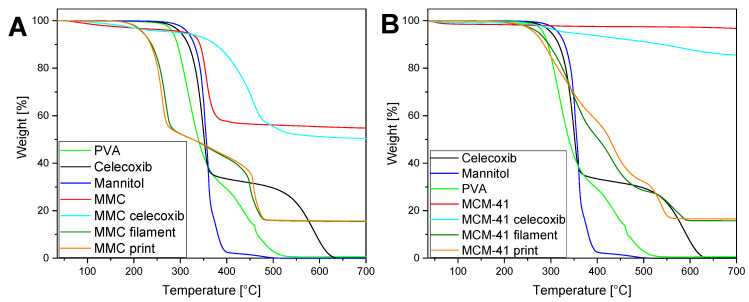
TGA thermograms of filaments, tablets, and individual components; (**A**) MMC-containing formulations; (**B**) MCM-41-containing formulations.

TGA was performed in order to investigate the thermal decomposition of the various materials and formulations, as well as to verify the degree of drug loading of celecoxib. The acquired thermograms are presented in Figure 6. Pure PVA starts to rapidly decompose at ~270 °C [64], while mannitol is observed to be stable at temperatures below 285 °C [65]. The trace for celecoxib shows a sharp, two-step mass loss beginning at ~250 °C. The curve of unloaded MMC initially reveals a slight decrease of ~5% in mass, due to adsorbed moisture that was not removed during the first heat cycle to 150 °C, and subsequently, a decomposition into MgO and CO_2_ at 370 °C [26]. In the case of the drug-loaded MMC, ~5% in mass due to moisture is also removed in the beginning; however, the decomposition into MgO takes place over a wider range of temperatures, starting at 330 °C and ending at a plateau at 530 °C. It is interesting to note that the loading of celecoxib into MMC appears to increase its thermal stability—an effect that may be beneficial for thermolabile drugs used in additive manufacturing processes requiring elevated temperatures. By comparing these two curves, the amount of celecoxib in the drug-loaded MMC may be calculated, since both samples consist solely of MgO after decomposition at the highest temperatures. The MgO remaining in the sample at the end of the TGA run is a certain fraction of the starting MMC material, and this fraction can be determined by comparing the start and end mass percentages in the unloaded MMC sample. Applying this same fraction to the MgO mass remaining at the end of the TGA run for the drug-loaded MMC sample will provide us with the amount of MMC in the sample. The difference between the starting sample mass and this value is the mass of the loaded drug. Specifically, the amount of MMC in the drug-loaded MMC sample is equal to the amount of MgO (from this sample) remaining at 700 °C multiplied by the ratio of the unloaded MMC amount at 270 °C (to exclude adsorbed moisture) to the corresponding amount of MgO after decarbonation at 700 °C. Thus, the mass percentage of MMC of the drug-loaded MMC sample, after desorption of moisture, is equal to 50.44% × 95.43/55.75 = 86.34%. The mass percentage of this sample at 270 °C is 95.4%; hence, the mass percentage attributed to celecoxib is the difference between these two values, equal to 9.06%, and in excellent agreement with the theoretical loading of 9.1%.

From Figure 6A, it can be seen that the TGA curves for the filament and print of MMC are almost identical, and follow a two-step process until reaching a plateau at ~500 °C. By comparing the curves to the TGA curves of the constituent materials, we can deduce that the first mass loss process is due to the PVA and mannitol, while the second is due to the decomposition of celecoxib-loaded MMC. Interestingly, the mass loss begins at ~190 °C, which is significantly lower than the onset temperature for mass loss in the PVA or mannitol curves. This can be expected, since the incorporation of the plasticizer mannitol in PVA enhances the molecular mobility of the blend, and has been shown to lower the glass transition temperature T_g_ of the mixture [66]. Note that, at 205 °C—the temperature used during the printing process—the mass loss percentage is relatively small, at only 2.24%. In the same manner as detailed above, the amount of MMC in the filament and print samples can be calculated from the TGA curves, based on the assumption that only MgO remains after heating the samples to 700 °C. In these samples, no initial significant mass loss due to moisture desorption was apparent and, thus, the starting plateau used for the calculations was at 160 °C. After heating to 700 °C, the mass was reduced to 15.65% for both samples, and the MMC mass percentage was calculated as 15.65% × 95.43/55.75 = 26.79%. Given that the percentage of drug-loaded MMC used in the extrusion was 30%, and of that figure 9.1% was celecoxib, the percentage of MMC in the extrusion process was 30% × 90.9% = 27.27%, corresponding very well to the value calculated from the TGA curves. 

Regarding the samples of the alternative mesoporous material, heating the raw MCM-41 sample to 700 °C should result in no decomposition or mass loss other than the desorption of moisture. Indeed, the unloaded MCM-41 samples exhibit a small mass loss of ~3%. The mass loss in the thermogram of the drug-loaded MCM-41 is attributed both to adsorbed moisture and to the amount of celecoxib loaded in the pores. Consequently, the drug loading percentage of MCM-41 is calculated as 96.78 − 85.62 = 11.16%, corresponding well with the theoretical drug loading of 9.1%. The MCM-41 filament and print exhibit a comparable behavior to the MMC filament and print, where the mass loss begins to occur at a lower temperature compared to mannitol or PVA alone, and rapidly increases at around 260 °C—an observation that can again be ascribed to the intermolecular interactions between mannitol and PVA [66]. Note that the mass loss of these samples is <1% at the printing temperature of 205 °C. The higher decomposition onset temperature of MCM-41 filament and print samples, compared to the MMC samples, may be attributed to the higher polymer-to-plasticizer ratio between the two: 55:25 in the MCM-41 samples and 45:25 in the MMC samples. After heating the filament and print samples of MCM-41 to 700 °C, the remaining mass percentage would correspond to the percentage of MCM-41 in these formulations. These values were found to be 15.75% and 16.56% for the MCM-41 filament and print, respectively. In the extrusion process, the percentage of drug-loaded MCM-41 used is 20%, of which 9.1% consists of celecoxib, and thus the percentage of pure MCM-41 in the mixture is 18.18%, in good agreement with the calculated values from the TGA curves.

### 3.7. SEM

**Figure 7:**
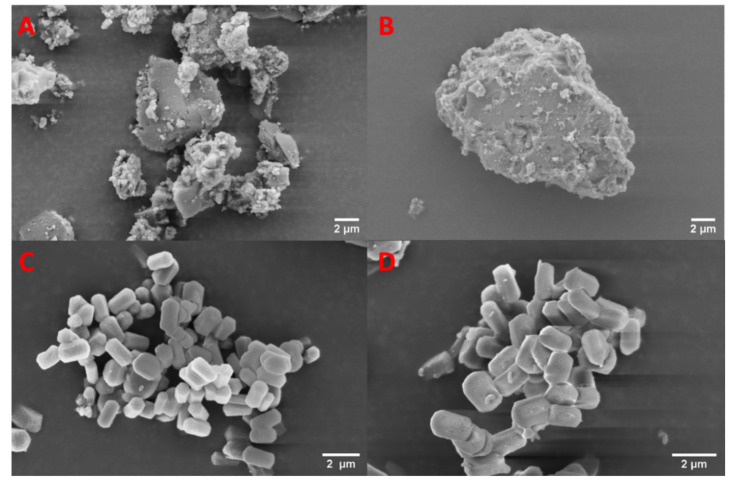
SEM images of (**A**) unloaded MMC; (**B**) drug-loaded MMC; (**C**) unloaded MCM-41; and (**D**) drug-loaded MCM-41.

The SEM images of the mesoporous materials before and after drug loading are presented in Figure 7. The hexagonal structure of MCM-41 is apparent, as is its small crystal size. In the case of the MMC, the aggregates of the nanoparticles vary in size from less than one micrometer to several tens of micrometers. The mesoporous materials show no discernable morphological differences after being loaded with celecoxib. 

**Figure 8:**
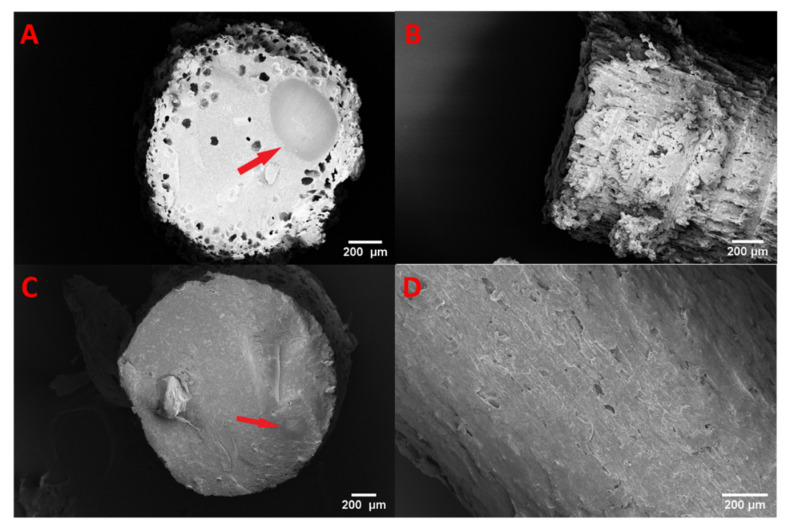
SEM images of mesoporous material containing filaments: (**A**) cross-section and (**B**) side view of MMC-containing filament; (**C**) cross-section and (**D**) side view of MCM-41-containing filament. The arrows indicate polymer inclusions.

The two different filament formulations were investigated, and images of the filament surface and cross-section are depicted in Figure 8. SEM images of the MMC-containing filament show a distinctively rough surface morphology compared to the filament containing drug-loaded MCM-41, which exhibits a relatively smooth surface with no discernable defects. The cross-section of both filaments reveals some degree of inhomogeneity of the ingredients, where an inclusion of polymer is surrounded by the molten physical mixture of the loaded mesoporous material bound together by the polymer in an outer shell (see Figure 8). This effect is more pronounced with a larger inclusion of polymer in the case of the MMC formulation.

**Figure 9:**
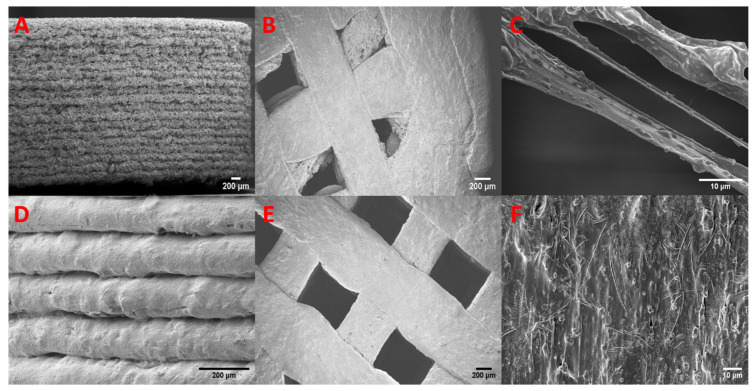
SEM images of printed tablets. Top row: MMC-T70 tablet: (**A**) side view; (**B**) top view; (**C**) imperfect fiber within the structure of the tablet. Bottom row: MCM-41-T70 tablet: (**D**) side view; (**E**) top view; (**F**) magnified view of the surface of the top layer.

The morphology of the printed tablets with 70% infill is shown in Figure 9. The tablets of both mesoporous materials exhibited high accuracy in reproducing the model used for printing, with the grid pattern being explicitly detailed in Figure 9B,E. A close inspection of the print—see Figure 9C,F—reveals the presence of mesoporous particles surrounded by molten polymer. This suggests a good distribution of drug-loaded material throughout the body of the tablets of both formulations. The side view of the MMC-containing tablet displays a fibrous-like structure, similar to the rough morphology of the MMC filament, which is likely due to the large range in size of the aggregates of MMC particles within the polymer. This effect is also noticeable in the top view of the tablet. The first layer is relatively smooth, as it was attached to the masking tape during the printing, but subsequent layers can be seen to have a rougher surface. In contrast, the side view of the MCM-41-containing tablet in Figure 9D shows a well-defined layered structure with a consistent height of ~0.145 mm, in good agreement with the parameters set in the printer, i.e., 0.15 mm. The top view of the tablet shows the smooth nature on both the first and subsequent layers of the print. 

### 3.8. In Vitro Drug Release

**Figure 10:**
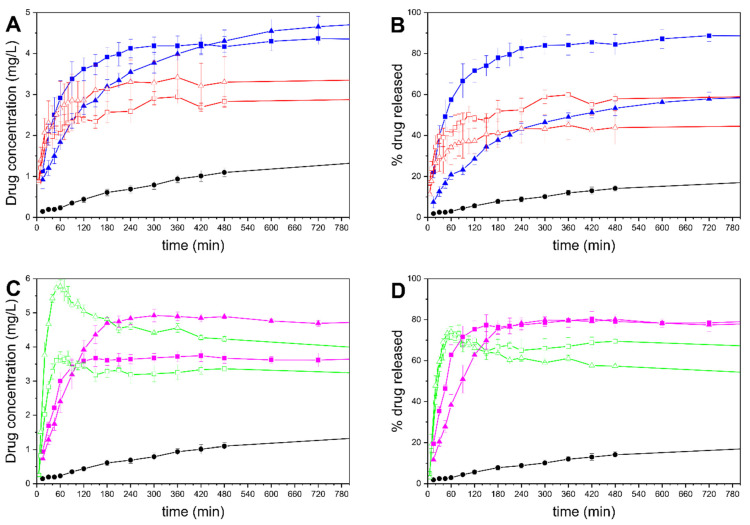
Drug release curves. (**A**,**B**): MMC formulations; (**C**,**D**): MCM-41 formulations. (**A**,**C**): concentration of drug released over time; (**B**,**D**): % drug released over time. Crystalline celecoxib: ●, MMC-50: □, MMC-70: 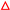, MMC-T50: ■, MMC-T70: ▲, MCM-41-50: □, MCM-41-70: 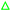, MCM-41-T50: ■, MCM-41-T70: ▲.

The in vitro drug release behaviors of the formulations are presented in Figure 10. The top panels show the release from the MMC tablets with 50 and 70% infill, compared to drug-loaded MMC and crystalline drug of an equal amount of celecoxib, presented as concentration and percentage of the drug in the phosphate buffer released over time, whereas the bottom row panels are associated with MCM-41 formulations. The 100% drug release value corresponds to the amount of the theoretical loading of each formulation.

In this study, the amounts of celecoxib used in all samples (see Table 1) were more than required to achieve the equilibrium solubility in 0.1 M phosphate buffer—which was measured to be 1.4 μg/mL ± 0.2, and matches well with previous research [48]. As expected, the drug release from drug-loaded MMC is significantly quicker and reaches a higher concentration and release percentage compared to the drug in its crystalline form [28]. The final concentrations for MMC-50 and MMC-70 were measured to be 2.82 and 3.3 μg/mL, respectively—more than double the measured solubility of celecoxib. The MMC-70 powder sample, which contained the higher celecoxib dose, reached a final release percentage of 46%, whereas the MMC-50 sample reached 61%. Although both samples achieved a supersaturated concentration, it is not certain whether the final release percentage being less than 100% was a result of some of the released drug precipitating rapidly after dissolution, or because some of the drug was not released from the particles. A previous study of celecoxib released from MMC particles showed both a supersaturation and subsequent precipitation release behavior, but in that study the drug loading was approximately 10 times greater than that used in the present investigation [28]. 

As for the tablets containing MMC, MMC-T50 outperformed MMC-T70 in terms of both rate of release and percentage of drug released. MMC-T50 exhibited a more rapid drug release, achieving ~80% after 4 h of stirring, after which the concentration stabilized, whereas MMC-T70 showed a significantly slower release for celecoxib, with the curve approaching a plateau only after 10–12 h. The percentages of drug released after 24 h were ~88% and ~63%, while the maximum concentrations achieved were 4.29 and 5.05 μg/mL, for MMC-T50 and MMC-T70, respectively; these values were both more than three times the equilibrium solubility of the drug. 

The two drug-loaded MCM-41 powder samples exhibit similar release profiles between them, with the higher dose in the MCM-41-70 sample leading to a correspondingly greater drug release compared to the MCM-41-50 sample. In both powder formulations, the release rate is significantly more rapid compared to the crystalline drug, as has been previously demonstrated [67]. MCM-41-50 and MCM-41-70 both reveal a supersaturation of celecoxib peaking at 3.7 and 5.7 μg/mL, respectively, followed by a quick decrease to approximately 3 μg/mL and 3.5 μg/mL for MCM-41-50 and MCM-41-70, respectively, as celecoxib recrystallizes and precipitates in the medium. The tablets containing drug-loaded MCM-41 for both infill percentages released ~80% of the loaded drug, with the MCM-41-T50 samples exhibiting a significantly faster release than the 70% infill tablets, and achieving the final concentration after only 120 min. The final concentrations of celecoxib were 3.8 and 4.9 μg/mL for the MCM-41-T50 and MCM-41-T70 tablets, respectively. As with the tablets containing MMC, there was no indication of these levels decreasing over time, which can be attributed to the presence of dissolved polymers that prevent recrystallization of celecoxib from the supersaturated state [27,68].

The different materials used in the tablets all contribute to the observed drug release profiles. For mesoporous materials such as MMC and MCM-41, the release is governed by a diffusion mechanism [21,25,26,69]. On the other hand, drug diffusion and polymer dissolution both contribute to the drug release from water-swellable and -soluble polymeric formulations [70]. Moreover, the release through PVA has been shown to be mediated by an erosion process [71,72]. Thus, it is suggested that in the case of both MMC and MCM-41 tablets, the drug release process is governed by a combination of the aforementioned mechanisms. The faster release in the 50% infill tablets compared to the 70% infill tablets can be attributed to the larger spaces in the tablet that allow for a higher degree of swelling while still offering access to the dissolution medium, as well as an increased surface area accessible to the dissolution media. This effect is further enhanced by the removal of the top and bottom layers of the tablets—see Figure 9B,E—allowing for faster access of the release medium to the tablet’s core. Furthermore, all formulations containing MCM-41 exhibited a quicker drug release compared to the corresponding formulations containing MMC. As stated earlier, the gas sorption results indicate that the drug loaded in MCM-41 is located close to the surface of the particles, which may contribute to the quicker drug release compared to both powders and tablets containing MMC.

## 4. Conclusions

In the current work, a novel combinatorial system for creating oral dosage forms was explored, comprising a drug-loaded mesoporous material and a polymer, formulated into 3D-printed tablets. Two mesoporous materials were studied, and showed the capacity to retain a loaded poorly soluble drug in an amorphous state after both hot-melt extrusion and 3D printing. In vitro drug release tests showed that the printed tablets produced higher drug concentrations and release percentages compared to the crystalline drug or the corresponding plain drug-loaded mesoporous materials. This novel approach, utilizing drug-loaded mesoporous materials in a printed tablet via FDM, shows great promise in achieving personalized oral dosage forms for poorly soluble drugs. 

## Figures and Tables

**Table 1:** Dosage of celecoxib within the formulations of MMC and MCM-41.

	Crystalline Celecoxib	MMC-50	MMC-70	MCM-41-50	MCM-41-70	MMC-T50	MMC-T70	MCM-41-T50	MCM-41-T70
Celecoxib dose (mg)	7.1	4.6	7.1	4.33	5.67	4.6	7.1	4.33	5.67

**Table 2:** SSA, pore volume, and pore width for unloaded and drug-loaded MMC and MCM-41.

	Specific Surface Area (m^2^/g)	Pore Volume (cm^3^/g)	Pore Width (nm)
MMC	242.9	0.44	6.84
MMC Celecoxib	187.5	0.37	7.40
MCM-41	1082.0	0.90	3.18
MCM-41 Celecoxib	840.9	0.68	2.95

**Table 3:** Properties of filaments and tablets containing MMC and MCM-41. Mean values and standard deviations are given for the samples.

	Mass ± St.D. (mg)	Height ± St.D. (mm)	Diameter ± St.D. (mm)
MMC Filament	-	-	1.60 ± 0.03
MMC-T50	173 ± 8	2.96 ± 0.06	11.03 ± 0.03
MMC-T70	264 ± 12	3.00 ± 0.01	11.11 ± 0.01
MCM-41 Filament	-	-	1.72 ± 0.02
MCM-41-T50	241 ± 7	2.99 ± 0.02	11.00 ± 0.01
MCM-41-T70	312 ± 8	3.00 ± 0.01	11.00 ± 0.04

## Data Availability

The data collected and its analysis used in this study are available from the corresponding author, following a request.
